# Foreign Medical Literature

**Published:** 1818-07-01

**Authors:** 


### THIRD DEPARTMENT.

### Foreign Medical Literature.

### I.

*On Inequilibrium in the Balance of the Circulation and Excitability, as the principal Source and Phenomenon in most Diseases.* Translated and condensed from the French of Vialle and Broussais.

The important doctrine of derangement of balance in the vascular and nervous systems, as the great cause of many diseases, both in the class Phlegmasiæ and Neuroses, begins to spread through France, and will, in all probability, produce a considerable revolution in the science of medicine. M. Broussais, the distinguished author of a work on *Chronic Inflammations,* [ Phlegmasies Chroniques ] has recently published a work, entitled "Examen de la doctrine medicale," in which he combats with great success, the pernicious doctrines of Brunonianism, which still retain a considerable ascendancy on the continent. M. Vialle is a disciple of Broussais, and has given an outline of his master's and his own doctrine in a work entitled, "Considerations generales sur l'Irritation, &c." which work shall form the basis of this article.

The human frame is endued with two powers, faculties, or properties, *sensibility* and *mobility,* to which all its phenomena may be referred, and of the *final* causes of which we are totally ignorant. The laws which govern them are the proper object of study. Under the term *vascular system* we comprehend not only those vessels which carry red blood, but all those concerned in exhalation, absorption, secretion, and excretion. These contain the various fluids which are seen in the body, and impress upon them divers movements necessary for organization and the support of life. They are therefore endued with sensibility and mobility. The fluids themselves are almost entirely passive, though they exert a considerable influence on the motive and sensible fibre, according to their qualities, quantities, changes, or combinations; and also according to the obstruction they may meet in their destined courses. The *nervous* system again is concerned in every sensation—every movement; in fact, it gives *origin* to them. These two systems enter into the composition of every organ. On their harmony depend the freedom of function and integrity of structure which characterize health; and every derangement in their balance tends to the production of disease. Life is manifested to the physiologist only by the exercise of sensibility and mobility; but this exercise is determined by various agents, which act externally or internally on the living machine, and the result has been termed excitation, excitement, stimulation, or irritation, which are all nearly synonimous, and but another name for life itself. Physicians have allowed that this excitement may rise to excessive, or fall to deficient action; both of which are aberrations from health. Haller uses these memorable words—"*In modica irritabilitate sanitas:—ejus defectus est torpor aut paralysis: excessus varii morbi.*" *Physiol. Tom.* 4.

I shall beg leave to substitute *irritation* for irritability, and proceed to consider it as, 1mo.—*Moderate,* or at the healthy standard; 2do.—*Augmented,* (super-irritation or excitement) or beyond the natural degree, giving rise to various ills; 3tio.—*Deficient,* (sub-irritation) attended with torpor or debility. But contrary to the Brunonian doctrine, I shall endeavour to prove that the states of excessive and deficient irritation [super-irritation or excitement, and sub-irritation or excitement] are hardly ever *general,* or completely isolated one from the other, but, on the contrary, co-exist, though in different organs.

1.—That moderate degree of irritation or excitement which corresponds to health, is susceptible of great variety, from idiosyncrasy of constitution—and, in fact, is always *individual.* If the various systems, organs, or functions were *equally* developed and proportioned to each other, then we should have that *imaginary* equilibrium which the ancients denominated, the *tempered temperament. General strength* would result from this equilibrium, and from a regular and energetic exercise of all the functions; *general weakness* would depend on a less energetic exercise of the same. But for the most part, we observe a *predominance* of some systems, organs, or functions over other systems, organs, or functions in the same individual. To understand this, it is necessary to recollect, that those stimuli or excitants which act on the moving and sensible fibres, cannot have a general or uniform action throughout the whole frame; cannot excite all the organs to the same degree. On the contrary, they more frequently act *partially*, or on certain sets of organs, affecting others remotely, or feebly by sympathy. *Irregular* excitement then must be the consequence of *inordinate* stimulation, which *irregularity* of excitement is still further increased by the well-known law, that *the more a part is irritated, the more susceptible it becomes of an increase of irritation.* This, of course, soon becomes a morbid state. There is another important law, which has never been disproved since the days of Hippocrates, namely: *the greater the degree of irritation or excitement in one part, the less there must be in some other.* Strength and debility then are rarely general; but are most frequently found in the same individual; a truth which saps at once the whole foundation of Brunonianism. It is very true, that an inequilibrium of this kind, to a certain extent, is not incompatible with health, and only shews itself in the shape of variety of temperament. But carried beyond this boundary, it passes into disease.

When we consider the influence of the various agents that surround us, air, food, drink, the passions, &c. on the human frame, we cannot but admit that their *excess and deficiency* must be constantly producing super or sub-irritation, in all degrees. But in either case, the result is derangement of the balance in the vascular system, evinced by irritation in some internal viscus, amounting to inflammation, fever, or internal hæmorrhage; or in the nervous system, giving origin to those various diseases arranged in the class *Neuroses.*

It will not be questioned that *excessive* stimulation may produce super-irritation or excitement, with consequent derangement of balance in the circulation; but some may be disposed to doubt whether *defective* stimulation can tend to the same event. We can admit, indeed, that a *general* and regular subduction of stimuli, as of food, drink, light, heat, &c. may induce *general* debility and emaciation, without deranging the balance of the circulation and excitability; for this is the very process which the physician is so often called upon to effect. But in common life, this is a rare occurrence; for, in nine cases out of ten, where there is *defective* stimulation of one kind or of one organ, there is *excessive* stimulation of some other kind or of some other organ; the consequence of which is *irregular* excitement. Nature herself, indeed, tends to induce irregular concentrations of excitability, and local accumulations of blood, in all morbid or excessive subductions of stimuli. The great viscera seem to attract to themselves the blood and vital energy, in all cases where the sum total of it is extremely reduced. Thus intense cold, while it stupifies, will produce internal inflammation; great degrees of hunger and thirst will determine gastritis; and we see, in cases of extreme exhaustion, the blood disappear from the surface to feed the interior organs, which, on dissection, are found gorged and congested.

Let us pause here for a moment, and make a few reflections on these important observations of the French physician. The experiments of Dr. Seeds, related in the first volume of the *Medico-Chirurgical Journal and Review*, corroborate the foregoing statements. Take, for instance, the sixth experiment, *page* 443, where the animal was bled to death, by opening the internal jugular veins. "The contents," says he, "of the *cranium and spinal canal were so gorged with blood*, that it might, at first sight, have been imagined that blood-letting would have saved the animal." In every case where the animals were bled to death, there was effusion of water on the brain and spinal cord, often accompanied by red spots like inflammation. Every one must also have seen effusion in the chest, from carrying blood-letting too far in certain kinds of pneumonia, accompanied by typhoid fever, with irregular distributions of blood. With these facts and reasonings before our eyes, is it not evident that the present rage for subduing fever by blood-letting *alone*, and that not by pints, but by *half-gallons* at a time, is pregnant with danger, and likely to bring a valuable remedy into utter disgrace?

In all those febrile states of the system, where the balance of the circulation and excitement is greatly deranged; where one organ is gorged and another exanguious; where one part is torpid and another in a high state of irritation, we shall err most lamentably in attempting the restoration of balance in the circulation and excitement, by *profuse* subductions of the vital fluid *alone*. The principle which directs this practice is a confined one, and rests on limited views of febrile commotion. The enlightened medical philosopher will assign venesection the foremost rank, it is true; but he will no more trust to that *alone,* than the nation would trust to a Wellington without soldiers. He will call in the assistance of those other remedies which experience has proved to be possessed of power *in equalizing the circulation and excitement.* Among these, *mercury,* both in its purgative and salivary character, holds the very next rank to blood-letting; after which follow the cold and warm affusion, antimony, diluents, &c. and even well-timed stimuli.*

But to return to our author. These irregularities of excitement will be the more easily induced, by inordinate stimuli, in proportion to the enfeebled state of the system generally; for it has always been observed, that the equilibrium of the functions is more easily broken, and that irregular concentrations of vital energy more frequently take place, when the sum total of strength is below par.

Although it is almost impossible that any one of the great internal viscera should continue long in a state of irritation without affecting others by contiguity or sympathy; yet we shall generally find a particular viscus the *principal focus* of irritation. This focus establishes itself most frequently in the *mucous membranes*—the most sensible tissues in the human frame, and which, in reality, form the organs of internal sense. To them are immediately applied the various *excitantia* from without [food, drink, &c. to those of the digestive apparatus; atmospheric air, with its various impregnations and degrees of temperature to the mucous tissues of the respiratory apparatus], and they are the centres of the greatest number of sympathies.

As these tissues are extremely vascular, the irritation does not long confine itself to the nervous portion of their structure; but, according to the well-known law, "*ubi stimulus, ibi fluxus,*" an inflammation or congestion —in some rare instances, hæmorrhage, is the result. The parenchymæ, and the serous membranes become affected in the same manner, primitively or consecutively. On the other hand, the *muscular* structures are more generally the seats of nervous *irritation* alone. This nervous irritation we see sometimes *exalted* to spasms, or convulsions; sometimes *diminished* to torpor, or even syncope or paralysis. In all these cases we plainly observe, that the

---

*See the excellent paper of Mr. Travers on Iritis, in the Bibliological Department.—Also, Armstrong's late work in the same.

more the vital energy is augmented, or as it were, accumulated in one part, the more it is reduced in others. *Overplus* of action in one organ, annihilates the *regular* action of another, as was long ago observed by Hippocrates, who did not see things through the light of Brunonianism.

It is very true, indeed, that at the commencement of diseases, and in strong subjects, with a vascular system well developed, we shall see a state of super-irritation or excitement in several parts or organs of the body, internal and external, at the same time. But it is equally true, that if this super-irritation or excitement runs very high, or makes much progress, it will presently precipitate itself on some of the vital viscera, and there play round a centre, to a greater or less extent. Then it is, we see the exterior of the body, and the less important organs enfeebled—the muscular force becomes prostrated, chained down, or, as it were, planet-struck, [syderées] excepting in some rare instances, where the muscles are the seat of irritation; and then we see an opposite train of phenomena in the viscera: they are, as for example, in tetanus, so *torpid* as to bear the most enormous stimulation without danger of inflammation—the most enormous cathartics, without the chance of purgation*.

2.—The irritation of an organ may determine, by sympathy, an irritation in one or several other organs at a distance. This is particularly remarkable in the mucous membranes, where, as observed by Bichat, a *single point* of irritation is capable of disturbing the action of other organs or systems, in all degrees, from the most trivial sympathetic ailment, up to delirium, tremors, convulsions, irregularity and intermission of the pulse, or death itself, from excess of pain. The seats of these sympathetic irritations are also suddenly transferred from point to point; and what is of still more importance, the irritations are liable to be suddenly converted into unequivocal inflammations—a strong proof that the *Neuroses*, the *Phlegmasiæ*, and the Hæmorrhagiæ are but modifications of the same affection. Thus irritation is the parent or first step

---

* This explains a circumstance so ably insisted on by Dr. Dickson—the necessity of active purgation in tetanus, and in its *precursory* state of torpor in the bowels. Strong purgatives, in fact, tend to restore the balance of the excitability, and the equilibrium of the circulation. This should be kept in view in all febrile states of the system, where super-irritation prevails in the vascular and nervous systems, while the secretory and digestive organs are torpid.—*Ed.*

to inflammation; for the former disturbs the balance of the circulation, and this disturbance increases the irritation, or leads to reaction; consequently the vascular and nervous systems act and re-act on each other, and any deviation from harmony between the two, leads to numerous diseases. Sometimes these irritations, and vaccillations in the balance of the circulation and excitement gradually subside spontaneously, for the most part accompanied by some *secretory evacuation* from the system, which restores the equilibrium.

3. Now notwithstanding we have shewn that *excess* and *deficiency* of excitement are very generally combined in the same individual, though existing in different organs, yet our attention is to be chiefly directed to the *former*, or super-irritation. Rarely indeed is *debility* or sub-excitement, a dangerous disease; rarely is it unaccompanied by some neighbouring irritation, as its cause. Hence the infatuated Brunonian, by applying stimuli in certain cases of debility, has too often hurried on the *concealed* super-irritation to a fatal disorganization! We should therefore be cautious how we use stimulation; and never venture on it, unless where there is strong evidence that *little or no super-excitement* is lurking about any vital organ internally.

4. *Diseases from excess of excitement.* Whenever *irritation* in an organ is carried beyond a certain limit, it constitutes disease, whether it be determined on the vascular, the lymphatic, or the nervous structure of those organs or parts. In the *first* case it produces inflammation or hæmorrhage; in the *second*, sub-inflammation; in the *third*, neuroses.

A. When the *sanguiferous* capillaries that enter into the parenchymatous structure of an organ are the seat of the irritation, phlegmasia or hæmorrhage is the result. These diseases are more readily determined by a neuro-sanguine temperament, a full development of sensibility, and particularly of that sensibility, denominated by Bichat, *Organic.* The hæmorrhagic disposition also depends on an idiosyncrasy in the exhalent vessels, which favours the effusion of their sanguineous fluids, with scarcely any sensation of pain. The phlegmasiæ, where the blood is arrested and accumulated in the irritated points, are by far the most frequent of occurrence, and pathologists were very imperfectly acquainted with their various shades, and the characteristic symptoms that attend them, till the labours of M. Broussais threw so much light on the subject, *and demonstrated that it is principally to inflammation in the mucous membranes, more rarely in the serous tissues or parenchymatous structures of the internal viscera, we owe those various fevers, hitherto denominated* IDIOPATHIC.

This important doctrine, indeed, was fast gaining ground, till the dogmas of Brown obscured the horizon of medical science. Sydenham, in imitation of Hippocrates, Areteus, Galen, &c. regarded the abdominal viscera as the principal seat of fever: and although he introduced false theories respecting putrescency, &c. his practice was good; in truth, it was what we are now coming round to, by the light of morbid anatomy. Dr. James Sims, more than 40 years ago, insisted that "the nervous and putrid fevers of Huxham and Pringle were owing to affections of the epigastric viscera; and that when fatal, there was frequently *an abscess in the brain*; but more frequently still, inflammation in the abdomen." He observes, that the reason why many recovered under the stimulating plan was, *because physicians were not always able to kill their patient by any means!*

The following memorable sentence of Baglivi may now be read with admiration of his sagacity: "Malignitatem *medicamentis calefacientibus* aggrediuntur, quibus non solum non submovetur, sed *latentes viscerum inflammationes,* quæ talium febrium ut plurimum sunt causæ genuinæ, magis magisque adaugentur."

B. Super-irritations in the vessels of the lymphatic system, whether absorbent, exhalent, or secerning, give rise to what M. Broussais denominates *sub-inflammations,* which may be either primitive, or, as is more generally the case, consecutive of irritation in the sanguineous capillary vessels. They are determined by the same causes, and in the same manner as these last. They are most frequently developed in those organs and structures where the lymphatic vessels predominate, or where they are enlivened by much vascularity or nervous fibrillæ. They are also frequently complicated with sub-inflammation of the blood vessel capillaries, as in scrofula, tubercular consumption, &c. *Super-irritation,* both of the sanguineous and lymphatic capillaries, besides causing phlegmasia and sub-inflammation, gives origin to other trains of phenomena not less important or interesting; such as augmentation or suppression, *i. e.* irregularity in the secretions, exhalations, and absorptions; hence result dropsies, colliquative perspirations, rapid emaciations, depravation of the secretions, exhalations and excretions, &c. To this source we may attribute those sudden augmentations of volume in some organs or parts, certain aneurismal enlargements of the heart, the formation of false membranes, &c.

C. When these *super-irritations* have their principal abode in the nervous system, they are classed under the head *neuroses.* But let it be borne in mind, that this system is never affected in an insulated manner; for whether the irritation be seated in those minute nervous expansions which are interlaced with the sanguiferous and lymphiferous vessels, or resident in the centres of the nervous cords which are nourished by the above-mentioned vessels, there is a constant participation of suffering in both systems. This is proved by the alteration of structure; even the disorganizations which result from long-continued nervous affections. There is not then, but a single degree; a single step from the neuroses to the phlegmasiæ. In the *first,* the irritation is *predominant* in the nervous system, and the phenomena are *chiefly* nervous; in the *latter* the irritation is *predominant* in the sanguiferous and lymphiferous systems, and the phenomena are *chiefly* vascular. In the *neuroses* the morbid phenomena consist mostly in simple lesion of function; while in the *phlegmasiæ,* these lesions of function are more quickly followed by deterioration of fluids, and disorganization of structure. *The more, however, the neuroses are viewed as in alliance with the phlegmasiæ, the more successful will be our treatment of them.* We must, in short, *materialize* the neuroses more than we have hitherto done, before we arrive at just notions respecting their nature and removal. The principal diseases of this class are, the neuralgiæ, convulsions, chorea, cramps, tetanus, epilepsy, mania, asthma, palpitations, syncope, spasms of the œsophagus, stomach, intestines; non-inflammatory pains in these organs, hypochondriasis, hysteria, nymphomania.

5. *Therapeutics.* It will readily be conceived that in diseases of *super-irritation,* our first object is to withdraw the stimuli which may have caused, and are still keeping up the excessive excitement. Thus extremes of heat and cold, light, &c. are to be avoided. The general mass of the circulation is to be reduced by bleeding, abstinence, and purgation. The febrile irritation on the surface, if any, is to be alleviated by cool air, ablutions, &c. Then counter-irritation is of the utmost consequence.

In the phlegmasiæ every source of irritation should be as much as possible withdrawn from the organs that are in a state of super-excitement. Simple and obvious as is this precept, it is not put in practice; for we every day see the abdominal viscera irritated by improper aliment or improper medicine in gastric and intestinal affections. General blood-letting is most useful where the parenchymatous structure of organs is the seat of irritation, and where the general vascular system is highly excited. Local bleedings, on the other hand, are more proper in the phlegmasiæ of the mucous membranes, and in all cases where the sanguiferous system is not much disturbed. Where the general vascular excitement is great, general should always precede local blood-letting; and the latter should be *copious*, otherwise it may increase instead of diminishing the local inflammation. The same remark is applicable to counter irritation. It should be preceded by general evacuations in all cases where there is strong vascular action. The chronic or sub-inflammations must be treated by evacuations and counter-irritations in the same way, with rigid abstinence.

In the class *Neuroses* infinite mischief is every day done by stimulation. The view which we have given above of the affinity between this class and the phlegmasiæ, will point out a more rational and successful method of treatment; namely, subduction of stimuli, evacuations, abstemiousness, counter irritation, and those means which restore the balance of the circulation and excitement. Any good that has ever resulted from the fœtid gums, stimuli, narcotics, &c. in the neuroses, is attributable to the operation of counter-irritation, as restorative of the balance of excitement.

---

We have preferred giving this detailed view of a most important doctrine, to the collection of a heterogeneons mass of curiosities or monstrosities, which would never bear a *second* reading. We venture to predict that this article will not only produce a considerable sensation in the medical world here, but have a salutary influence on the treatment of disease, when the spirit that conceived, and the hand that transcribed it, shall have fled to the skies, or mouldered in the tomb!

